# Diagnosis of Subungual Glomus Tumors with 18 MHz Ultrasound and CDFI

**DOI:** 10.1038/s41598-020-74774-7

**Published:** 2020-10-20

**Authors:** Li Chen, Yi-Hui Gao, Jie Chen, Yi-Jing Yao, Rui Wang, Qian Yu, Bing Hu, Li-Xin Jiang

**Affiliations:** Department of Ultrasound in Medicine, Shanghai Jiaotong University Affiliated Sixth People’s Hospital, Shanghai Institute of Ultrasound in Medicine, Shanghai, China

**Keywords:** Ultrasonography, Skin diseases

## Abstract

To evaluate the imaging features of subungual glomus tumors using 18 MHz high-frequency ultrasound with CDFI (Color Doppler Flow Imaging). 20 patients treated by surgical resection and examined by ultrasound between January 2008 and December 2019. All eligible cases are divided into two groups: Group A used the probe frequency of 9–14 MHz from January 2008 to December 2014, and Group B used the probe frequency of 18 MHz from January 2015 to December 2019. Patient demographics, clinical records, pathologic specimens and sonography features were reviewed. 50% of tumors in Group A and 100% of tumors in Group B showed clear boundary and regular shape. Blood flow signals were identified inside 50% tumors in Group A (3 in 6), all 14 cases with blood flow signals detected in Group B (14 in 14,100%). 2 cases were misdiagnosed and 1 case escaped diagnosis in Group A, no case was misdiagnosed in Group B. The accuracy of diagnosis rate of Group B is significantly higher than that of Group A. 18-MHz ultrasound combined with CDFI may be a practical useful tool for detecting subungual glomus tumors. More importantly 18-MHz ultrasound can obviously improve the diagnostic accuracy.

## Introduction

Subungual glomus tumors are often diagnosed by physical examination and classic symptoms: pain, tenderness and cold sensitivity^1^. However, because subungual glomus tumors are under the nail plate we cannot detect them by ocular inspection or palpation. It is easy to delayed the diagnosis or be misdiagnosed. Additionally, even if diagnosed correctly, a subungual glomus tumor operation with inaccurate tumor position and size measurement may result in incomplete surgical excision, leading to recurrence^2–4^. It is important to identify the location and extent of the subungual tumor before surgery. Preoperative ultrasound is capable of identifying very small subungual glomus tumor^5^. CDFI technology is able to increase the specificity of sonography by providing real-time evaluation of vascularity^6,7^. The combination of the high frequency ultrasound and CDFI technology can significantly improve the detection of glomus tumor.

## Materials and methods

### Patients and clinical data

We retrospectively searched the ultrasonic and pathologic databases using the search term “subungual glomus tumor” between January 2008 and December 2019. Inclusion criteria were patients who received the pathologic diagnosis based on surgical resection and with the diagnosis of subungual glomus tumor on ultrasonic imaging. Exclusion criteria were patients who lacked ultrasonic examination and who lacked a final diagnosis of subungual glomus tumor on gross specimens. Even if patients had surgery in our hospital with outside hospital images available for review, these patients were also excluded. All eligible cases are divided into two groups according to whether to use the 18 MHz probe: Group A used the probe frequency of 9–14 MHz from January 2008 to December 2014, and Group B used the probe frequency of 18 MHz from January 2015 to December 2019. Patient demographics, clinical records, pathologic specimens and sonography features were reviewed.

### Ethical approval

The patients’ sonography features data and clinical records in this study are not publicly, but we confirm that all methods were carried out in accordance with relevant guidelines and regulations and all experimental protocols were approved by the Institutional Ethics Committee of Shanghai Jiaotong University Affiliated Sixth People’s Hospital. We confirm that informed consent was obtained from all subjects or, if subjects are under 18, from a parent and/or legal guardian.

### Sonographic evaluations

All sonograms were reviewed by two fellowship-trained musculoskeletal radiologist with 5 and 10 years of experience retrospectively. All lesions were accurately investigated, storing several pictures and videos. The tumors were characterized by sonography according to their echogenicity, shape, location, size, margins, bone erosion, internal and peripheral blood flow signals.

All tumors had been scanned using the latest scanners at the time with high frequency transducers (9 MHz or more). Ultrasound diagnosis equipment included Toshiba Aplio500 Ultrasound System and Hitachi HI VISION Ascendus equipped with an 18-MHz high frequency transducer since January 2015. We used Siemens ACUSON Sequoia 512 equipped with a 8–14 MHz linear array probe and Esaote My Lab 60 equipped with a 9–14 MHz linear array probe from January 2008 to December 2014.

### Statistical analysis

According to the results of pathological diagnosis, the accuracy of ultrasound diagnosis of tumors from January 2008 to December 2013 and from January 2014 to December 2019 were calculated.

Differences were analyzed using the Student t-test, and Chi square χ^2^ test. All analyses were performed using SPSS software (version 18.0, SPSS Inc. Chicago, IL). The level of significance was set at P < 0.05.

## Results

### 2D and color Doppler ultrasonography

**Figure 1:**
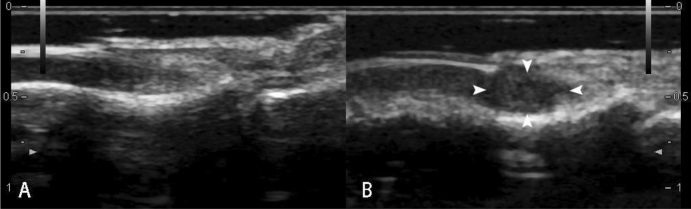
Sonography shows normal nail of the right ring finger (**A**). Arrow heads point to isoechoic Subungual glomus in the left ring finger (**B**).

**Figure 2:**
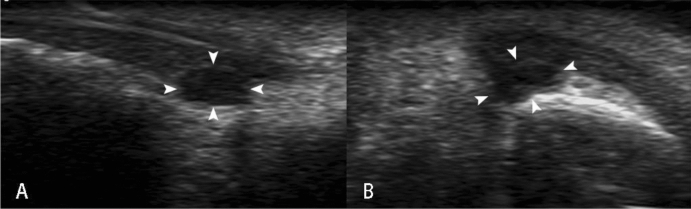
Arrow heads point to the hypoechoic Subungual glomus on long axis view (**A**) and short axis view (**B**) of the right thumb.

**Figure 3:**
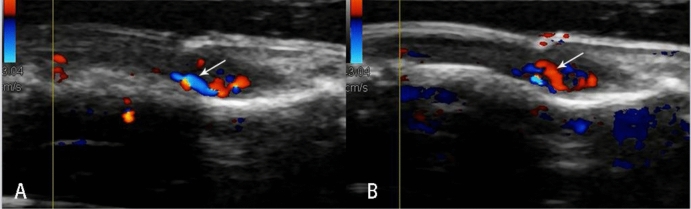
Color Doppler imaging shows abundant blood flow signals in the Subungual glomus. Arrows note the afferent artery (**A**, blue one) and the efferent vein (**B**, red one).

**Figure 4:**
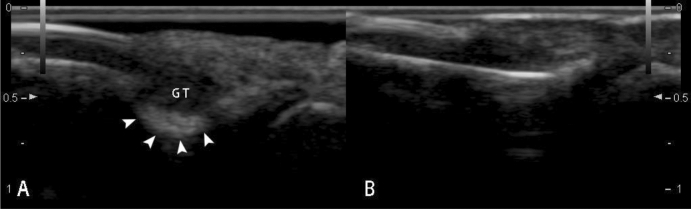
19-year-old female with painful nodule below the right index finger. Sonography shows a hypoechoic nodule (5.1 mm in length and 2.6 mm thickness) in the subungual region, four arrow heads point to erosion of distal phalanx (**A**). Sonography shows the normal cortex of distal phalanx in the contralateral index finger (**B**).

20 pathologically confirmed with ultrasound performed cases of subungual glomus tumor were collected in this study. The mean patient age was 35.5 years (range 19–61 years); 90% patients (18 in 20) were female and were 10% (2 in 20) male. The diameters of the longitudinal axis were between 3.1 and 9.4 mm (mean ± standard deviation 5.6 ± 2.0 mm). The diameters of the depth axis were between 2.1 and 6.4 mm (mean ± standard deviation 3.5 ± 1.3 mm). All lesions were hypoechoic or less isoechoic (Fig. 1), none were hyperechoic. The typical ultrasonography of glomus tumors was presented as solid hypoechoic nodules, regular oval shape (Fig. 2), internal abundant flow signals (Fig. 3) and peripheral bone erosion (Fig. 4). In this study, Group A including 6 cases used the probe frequency of 9–14 MHz from January 2008 to December 2014, and Group B including 14 cases used the probe frequency of 18 MHz from January 2015 to December 2019. 50% of tumors (3 in 6) in Group A and 100% of tumors in Group B showed clear boundary and regular shape. Blood flow signals were identified inside 50% tumors in Group A (3 in 6), all 14 cases with blood flow signals detected in Group B (14 in 14,100%) (Tables 1 and 2). 12 cases with rich blood flow and 2 with little blood flow were observed in Group B. The afferent artery and the efferent vein clearly noted in Group B in Fig. 3. 50% of all tumors (10 in 20) showed erosion of distal phalanx, the 10 cases were all in Group B, no case showed bony erosion in Group A. In sonography of these patients, the maximum length of these tumors with bone erosion were all greater than 5.0 mm (mean ± standard deviation 7.1 ± 1.8 mm), anteroposterior diameter of these tumors with bone erosion were all greater than 2.6 mm (mean ± standard deviation 4.27 ± 1.4 mm) (Tables 1 and 2).

**Table 1:** Demographic data and sonographic findings for group A.

			Axis (mm)			Internal	Internal	Bony
Case	Sex	Age (y)	Longitudinal	Depth	Boundary	Echogenicity	Vascularity	Erosion
1	F	30	4.8	3.0	Fuzzy	Isoechoic	P	N
2	F	43	3.4	2.1	Clear	Hypoechoic	N	N
3	F	61	6.2	3.2	Fuzzy	Isoechoic	P	N
4	F	45	3.2	2.3	Clear	Hypoechoic	N	N
5	F	25	4.3	2.2	Clear	Hypoechoic	P	N
6	F	21	N/A	N/A	N/A	N/A	N/A	N/A

F = female; M = male; P = positive; N = negative; N/A = not applicable.

Case 6 escaped diagnosis. Case 2 and 3 were misdiagnosed.

**Table 2:** Demographic data and sonographic findings for group B.

			Axis (mm)			Internal	Internal	Bony
Case	Sex	Age (y)	Longitudinal	Depth	Boundary	Echogenicity	Vascularity	Erosion
1	F	28	4.2	2.3	Clear	Hypoechoic	P	N
2	F	26	9.3	4.8	Clear	Hypoechoic	P, Rich	P
3	F	27	7.3	4.8	Clear	Hypoechoic	P	P
4	M	24	3.1	2.2	Clear	Hypoechoic	P, Rich	N
5	F	19	3.7	2.5	Clear	Hypoechoic	P, Rich	N
6	F	60	9.4	6.4	Clear	Hypoechoic	P, Rich	P
7	F	54	6.0	3.4	Clear	Hypoechoic	P, Rich	P
8	F	51	3.7	3.1	Clear	Hypoechoic	P, Rich	N
9	F	27	5.7	4.3	Clear	Hypoechoic	P, Rich	P
10	F	29	6.1	4.0	Clear	Hypoechoic	P, Rich	P
11	F	30	7.2	3.3	Clear	Hypoechoic	P, Rich	P
12	M	58	5.0	2.7	Clear	Hypoechoic	P, Rich	P
13	F	33	9.4	6.4	Clear	Hypoechoic	P, Rich	P
14	F	19	5.1	2.6	Clear	Hypoechoic	P, Rich	P

F = female; M = male; P = positive; N = negative.

## Discussion

As a rare benign tumor of the glomus body, glomus tumors can occur in various parts of the body especially at the end of the limb, but most commonly appear in subungual region of the fingertips, because the glomus bodies are particularly concentrated in this region^8–10^. Subungual glomus tumors affect women more than men^10^, our research showed a stronger female predominance than ever before research. The subungual glomus tumor especially affects the quality of life, it will cause the distal phalangeal bone erosion when the tumor volume increasing^2^, so early diagnosis and treatment have great significance. Subungual glomus tumors are often diagnosed by their classic triad symptoms, which include pain, tenderness and cold hypersensitivity^1^. However, owing to the occult site, they are rarely palpable. It is difficult to detect their exact locations and small sizes clinically. In cases of small glomus tumors, they are sometimes diagnosed incorrectly or misdiagnosed^10^.

**Figure 5:**
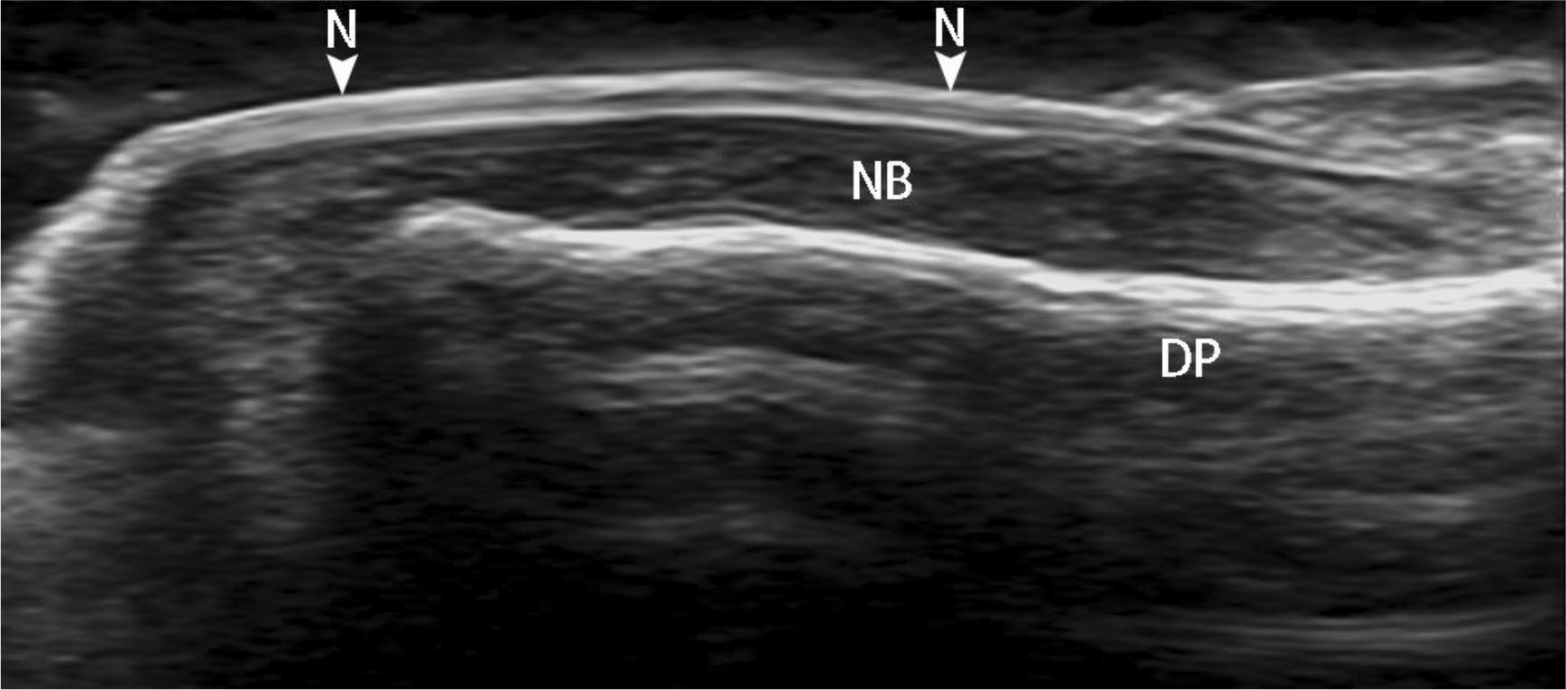
High resolution (frequency) ultrasound shows the normal dorsal anatomy of thumb, DP = cortex of distal phalanx, N = nail, NB = nail bed.

With advances in transducer technology, high frequency ultrasound has advantages in imaging of superficial tissues such as nails^11^, because of its physical characteristics. It can distinguish various anatomical structures of nail (Fig. 5) and surrounding tissues, and display the characteristic ultrasound manifestations of tumors, psoriasis, cysts, vascular malformations and other diseases around nail^11,12^. At present, there are more and more researches on nail disease sonography, such as in psoriasis, ultrasonography is commonly used to monitor disease progression^12–14^. Similarly, High frequency ultrasound can be used to diagnose and localize the subungual glomus tumor. In previous study, tumors < 2 mm in diameter can be identified in high-variable frequency ultrasound^15^.

Subjects in our research were divided into two groups: Group A including 6 cases used the probe frequency of 9–14 MHz from January 2008 to December 2014, and Group B including 14 cases used the probe frequency of 18 MHz from January 2015 to December 2019. The accuracy of ultrasound diagnosis in Group A was similar to that of MRI and ultrasound reported in previous articles. With the use of 18 MHz ultrasound probe, the boundaries of these tumors were clearly displayed, the accuracy of diagnosis rate of Group B (100%) is significantly higher than that of Group A. Our research found that 18 MHz US identified all subungual glomus tumors and their exact locations in the 14 patients evaluated. Higher than the frequency (9–14 MHz) used in the previous literature for the diagnosis of subungual glomus tumors, 18-MHz high-frequency US can significantly improve the diagnostic accuracy of subungual glomus tumor. Because the inner parts of the subungual glomus tumor could be clearly identified by 18-MHz high-frequency US. Without affecting the use of CDFI technology, the low-speed blood flow in subungual glomus tumor still can be clearly observed by18-MHz high-frequency US. Meanwhile, as a double-edged sword, ultrasonic frequency is higher, the resolution is better and the attenuation is more serious. 20 to 50 MHz high-frequency US is reported not adequate in imaging tumors of the nail unit as accessibility remains too superficial^11^. At present, there is no literature report on the application of 18 MHz in the diagnosis of subungual glomus tumor. Based on our research, we believe that 18 MHz is particularly suitable for the diagnosis of subungual glomus tumor and is worth spreading widely.

MRI and ultrasound can detect glomus tumors as small as 3 mm in diameter, CDFI can identify glomus tumors as small as 2 mm in diameter^5,13^. Intranodular vascularization is always present in glomus tumors^4^. Instead of PDI, CDFI is more suitable for the diagnosis of subungual glomus tumor which can show the direction of blood flow. Because glomus tumor often contains afferent and efferent blood vessels (feeding artery and efferent vein)^4^, and this typical feature clearly showed on CDFI in Group B in our study (Fig. 4).

It is reported that Color Doppler ultrasonography exhibited a 100% detection rate in digital glomus tumors^5^. Our series showed that only 50% of tumors had internal visible blood flow in Group A. With 18 MHz US, internal blood flow signals were identified in 100% subungual glomus tumors in Group B. The major reason may be the frequency of probe. Subungual glomus tumors were superficial position of glomus tumors, the higher frequency is better to show blood flow inside of the tumors on CDFI, may further influence the accuracy. On other hand, Color Doppler setting conditions were important, including minimum wall filter, low pulse repetition frequency, and high color gain. A big amount of gel is needed. Hand pressure was always minimized to avoid too much vessels compression^16–18^.

As an angiography, CDFI is a perfect tool for the diagnosis of small subungual glomus tumor especially for the fuzzy and isoechoic lesions. Intranodular vascularization has considerable value in differential diagnosis^6,7,17^. CDFI with 18 MHz US can support and improve upon the clinical diagnosis in subungual glomus tumor. Chiang^11^ reported that ultrasound with CDFI has a 100% identification rate for subungual glomus tumor, with complete resection for no long-term recurrence at 6 years after surgery.

Generally, ultrasound is not used in bone imaging, because of the high acoustic impedance of bone. Ultrasound cannot penetrate the bone cortex, and the internal structure of the bone manifested as shadow. But structure of the bone surface displayed as smooth linear hyperechogenicity very clearly on sonography. Such as bone erosion, uneven bone cortex, and periosteal reaction, the bone cortex changes can be observed by ultrasound. Ultrasonic features of joint bone erosions showed the high specificity and sensitivity^19^. Perpendicular to the acoustic beam, the distal phalanx erosions displayed more clearly than joint bone erosions on sonography. 50% of tumors (10 in 20) clearly presented erosion of distal phalanx in our study. The subungual space is very thin (approximately 1–2 mm thick, Fig. 1), any tumors arising within this area can result in erosion of the underlying bone. Phalangeal bony erosion is considered to depend on tumor size. We also found that when anteroposterior diameter of the tumor is more than 2.6 mm, it is often accompanied by different degrees of bone erosion which can be clearly displayed on the ultrasound image (Fig. 5). High frequency ultrasound showed the high specificity and sensitivity in detecting the erosion of the distal phalanx which caused by subungual glomus tumors. Surprisingly, none of the 20 cases with bone erosion is positive on X-ray report. US was considerably more sensitive than X-ray in detecting these bony erosions^10^. Maybe the radiologists did not consider entertain the prospective diagnosis of subungual glomus tumor on the radiologic reports of X-ray.

In our hospital, with the increasing accuracy of ultrasound diagnosis of subungual glomus tumors, more clinicians are apt to choose ultrasound as the first imaging method. The improvement of the accuracy of diagnosis is also related to the recognition of the radiologist to the glomus tumor. The visible lesions with characteristic symptoms probably do not benefit from US. However, the smaller tumors are easy to be detected with high frequency ultrasound, CDFI can show the blood flow signals in glomus tumor. The combination of 18 MHz-US and CDFI can significantly improve the detection rate of glomus tumor, and help to accurately detect exact locations and sizes of the subungual glomus tumors before surgery. Diagnosis of subungual glomus tumor with 18 MHz-US and CDFI is useful and worth spreading widely.

## Conclusions

The combination of 18 MHz-US and CDFI can significantly improve the detection rate of glomus tumor, and help to accurately detect exact locations and sizes of the subungual glomus tumors before surgery. Diagnosis of subungual glomus tumor with 18 MHz-US and CDFI is useful and worth spreading widely.

## Data Availability

The datasets generated during and analyzed during the current study are available from the corresponding authors on reasonable request.
